# A Rare Case of Life-Threatening Multicompartmental Spontaneous Intracranial Hemorrhage From a Grade 1 Convexity Meningioma

**DOI:** 10.7759/cureus.19178

**Published:** 2021-11-01

**Authors:** Mohammad Abolfotoh, Grzegorz Brzezicki, Peter Fiester, Daryoush Tavanaiepour

**Affiliations:** 1 Neurosurgery, University of Florida Health, Jacksonville, USA; 2 Neuroradiology, University of Florida Health, Jacksonville, USA

**Keywords:** subdural hematoma, bleeding inside meningioma, intracerebral hematoma, grade 1 meningioma, hemorrhagic meningioma

## Abstract

Meningiomas are slowly growing benign tumors. The incidence of hemorrhage associated with intracranial meningiomas is in the 0.5%-2.4% range. However, intracranial meningiomas with hemorrhagic presentation are associated with higher rates of overall major morbidity (36%) and mortality (21.1%). We report a case of a convexity meningioma presenting with intraparenchymal hematoma and bilateral acute subdural hematomas (SDH) in a comatose patient (Glasgow Coma Scale (GCS) score: 7) who had a history of recurrent episodes of headaches over the past few months.

Hemorrhagic presentation of a meningioma is a rare but potentially devastating event. Early recognition of the potential underlying meningioma as a cause of bleeding followed by rapid appropriate additional imaging is crucial to direct treatment plans to achieve the best outcome.

## Introduction

The majority of World Health Organization (WHO) grade 1 meningiomas are slowly growing benign tumors, while higher-grade meningiomas may have a more rapid rate of progression. Meningiomas typically have an insidious onset of presentation and rarely present with altered mental status (AMS). Although meningiomas are frequently vascular tumors, benign intracranial meningiomas do not typically hemorrhage. The incidence of hemorrhage associated with all types of intracranial meningiomas is in the 0.5%-2.4% range [1-3]. However, intracranial meningiomas with hemorrhagic presentation are associated with higher rates of overall major morbidity (36%) and mortality (21.1%) [2]. For patients who were unconscious on presentation, the overall mortality rate was 74.1%; however, the mortality rate among this group was decreased to 46.2% in surgically treated patients [2]. We report a case of a convexity meningioma presenting with intraparenchymal hemorrhage (IPH) and bilateral acute subdural hemorrhages in a comatose patient.

## Case presentation

**Figure 1:**
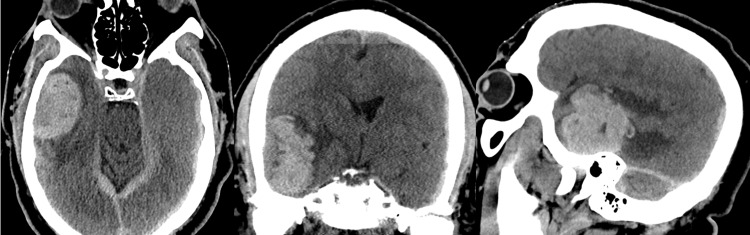
Multiplanar non-contrast head CT in a sexagenarian male presenting with progressive confusion and loss of consciousness. CT demonstrating a large, dural-based hemorrhage at the right temporal convexity with bilateral tentorial and hemispheric subdural hematomas and parafalcine subdural hematoma. Regional mass effect with right to left subfalcine shift is also evident.

**Figure 2:**
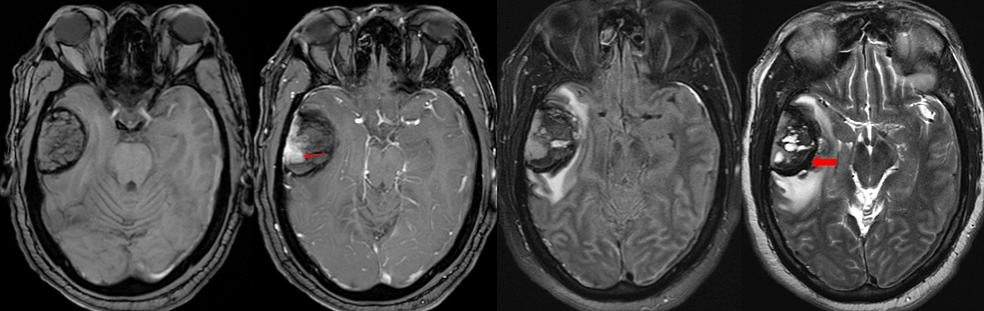
Initial pre- and post-contrast MRI (left to right: T1 pre- and post-contrast axial sequences, FLAIR sequence, and T2-weighted sequence). MRI demonstrating an extra-axial, dural-based mass at the right temporal convexity with nodular, dural-based enhancement (red arrow) with internal hemorrhagic cysts with variable age blood products. Multiple internal hemorrhagic cysts with fluid-fluid levels are present in addition to a well-defined and thickened T2WI hypointense capsule (red block arrow). The absence of a surrounding CSF cleft with prominent, perilesional vasogenic edema, mass effect, and right uncal herniation is also noted.

The patient is a 61-year-old male with unknown past medical history who presented to the emergency department with rapidly progressive confusion, followed by loss of consciousness, with no history or signs of trauma. On the initial evaluation, the patient had a Glasgow Coma Scale (GCS) score of 7, was localizing with bilateral upper extremities, and had a normal pupillary response. The patient was intubated for airway protection. Reformatted, multiplanar non-contrast head computed tomography (CT) demonstrated a large, dural-based hemorrhage at the right temporal convexity with bilateral tentorial and hemispheric subdural hematomas (SDH) and parafalcine subdural hematoma. There was also significant vasogenic edema surrounding the intraparenchymal hematoma (Figure 1). Brain magnetic resonance imaging (MRI) demonstrated an extra-axial, dural-based mass at the right temporal convexity with nodular, dural-based enhancement with internal hemorrhagic cysts with variable age blood products. Multiple internal hemorrhagic cysts with fluid-fluid levels were present in addition to a well-defined and thickened T2-weighted images (T2WI) hypointense capsule. The surrounding cerebrospinal fluid (CSF) cleft along the periphery of the mass was absent, and a prominent, perilesional vasogenic edema, mass effect, and uncal herniation were also noted (Figure 2). Head CT angiography (CTA) was negative for vascular abnormalities, and chest, abdomen, and pelvis CT were negative for primary and metastatic diseases.

**Figure 3:**
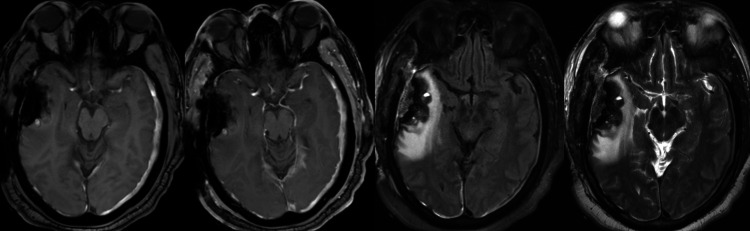
Pre- and post-contrast MRI status post-tumor resection (left to right: T1 pre- and post-contrast axial sequences, FLAIR sequence, and T2-weighted sequence). MRI demonstrating no evidence of residual enhancing tumor at the operative site with involution of the thickened, tumoral capsule and overall decreased mass effect.

An initial differential based on imaging and clinical scenario included a hemorrhagic metastatic lesion, a primary intra-axial tumor, a hemorrhagic meningioma, and a large, cavernous malformation. The patient’s neurological examination improved significantly over the ensuing days with high-dose steroids and supportive measures. Repeat head CT demonstrated stable right temporal intraparenchymal hemorrhage (IPH) and bilateral subdural hematomas (SDH). Subsequently, the patient was extubated. The patient and his brother gave a history of an episode of confusion few months prior, which resulted in hospital admission and a diagnosis of intracranial hemorrhage. The patient was discharged home with outpatient follow-up, but he failed to follow through. The patient also reported a four-month history of daily headaches self-medicated with aspirin products and poorly controlled hypertension. Semi-elective craniotomy was planned for the excision of the lesion and evacuation of the associated hematoma. The patient underwent typical navigation guided right temporal craniotomy. Upon opening of the dura, a soft, suckable, dural-based lesion was immediately identified. The frozen section was consistent with a dural-based tumor, likely a meningioma. Adjacent and deep to the tumor, there was a layer of acute hematoma, followed by a well-organized layer of multicompartmental chronic hematoma and then a very firm and organized fibrous capsule tightly adherent to the temporal lobe. The acute hematoma and well-organized chronic blood products were evacuated with suction and irrigation. The firm adherent capsule appeared to be macroscopically devoid of tumor tissue and was left behind to protect the normal brain. The dura itself appeared thickened and was resected within the edges of craniotomy. The subdural hematoma membrane was also resected within the craniotomy. The patient tolerated the procedure well and continued to recover without neurological deficits. Brain MRI following tumor resection demonstrated no evidence of residual, enhancing tumor with involution of the thickened, tumoral capsule and overall decreased mass effect (Figure 3). Permanent pathology revealed WHO grade I meningothelial meningioma. 

## Discussion

The overall intratumoral hemorrhage rate for brain tumors is 14.6% with a marked difference in hemorrhage rate between different histological types of tumors. Among primary brain tumors, mixed oligodendroglioma/astrocytoma is associated with the highest rate of hemorrhage (29.2%), followed by high-grade astrocytoma (12.5%), and then low-grade astrocytoma (10.9%). Melanoma is the most common hemorrhagic brain metastasis, with up to 50% presenting with hemorrhage [3].

Intracranial hemorrhage associated with meningioma is rare; however, it is associated with high mortality rates (ranges 28%-50%) [2]. Similar to our patient presentation, 57.1% of patients with hemorrhagic brain tumors present with an acute clinical syndrome [3]. Meningioma-associated hemorrhage can present in any intracranial compartment such as intratumoral, intraparenchymal, subarachnoid, and subdural [1]. The mechanism of bleeding has been postulated by multiple authors, including rupture of abnormal tumor vessels, extensive intratumoral infarct or necrosis, and vasoactive mediators released by the meningioma itself [1,2,4,5]. Subdural hematoma is not uncommon and presents in 49.2% of hemorrhagic convexity meningiomas such as the presented case [2]. The specific mechanism of subdural hemorrhage remains largely unclear. Some authors have suggested stretching of the subdural veins caused by tumor expansion ultimately leading to rupture [6], but this mechanism fails to explain large subdural hematomas coexisting with small meningiomas [7]. Our case is extremely unique in terms of its presentation with intratumoral, intraparenchymal, and diffuse bilateral subdural hematomas. Based on our intraoperative observation and clinical history, we hypothesize that the patient likely suffered from multiple silent hemorrhages into the tumor, followed by more significant episodes four months prior to this presentation and ultimately the most significant multicompartmental presenting hemorrhage. We suggest that the subdural hematoma was caused by the leakage of intratumoral hemorrhage into the subdural space due to the disruption of the leptomeningeal attachment of the tumor by the prior bleeding event. The hemorrhage was likely accentuated by the patient's aspirin use due to chronic headaches.

The risk factors of bleeding from intracranial meningiomas include the age of >70 and <30 years old, convexity or intraventricular meningioma, and the histopathological subtype of the meningioma: meningothelial, malignant, fibrous, and angioblastic [2,8]. Additionally, some other factors are proposed to potentially increase the risk of hemorrhage associated with meningiomas, such as hypertension, antiplatelet or anticoagulation therapy, traumatic brain injury, serotonin-modulating therapy, and high-dose estrogen replacement, although the exact causative mechanism is unknown [9-11]. The potential risk factors present in our patient include hypertension, aspirin use, convexity location, and histopathology consistent with WHO grade I meningothelial meningioma.

The main prognostic factors affecting the outcome after gross bleeding from meningioma are the patient's consciousness on presentation and surgical intervention [2,11]. Bosnjak et al. (2005) found that early recognition of the true nature and cause of the bleeding will affect the diagnostic workup, treatment, and ultimately outcome [2]. Conscious patients with early recognized hemorrhagic meningioma who underwent resection had a 96.2% survival rate [2]. In a very recently published article, the authors concluded that hemorrhagic meningiomas can be missed or misdiagnosed, while the main misdiagnoses were subdural hematoma (27.3%) and traumatic hematoma (13.6%) [12]. Our patient presented with right temporal IPH, bilateral SDH, and extensive vasogenic edema in the right temporal lobe that warranted brain MRI with and without contrast and lead to the discovery of the underlying lesion. As a result, the patient was started on high-dose dexamethasone with rapid improvement of neurological examination. Furthermore, the clinical improvement changed the treatment plan from emergency craniotomy for the evacuation of intracerebral hematoma to semi-elective craniotomy for the excision of hemorrhagic brain tumor with the avoidance of the potential significant postoperative cerebral edema as a result of pretreatment with steroids. Additionally, the MRI results and clinical improvement allowed us to make an intraoperative decision to forego unnecessary resection of organized thick adherent capsule risking significant injury to the temporal lobe after we felt that we achieved gross total resection and the frozen section suggested a low-grade meningioma.

## Conclusions

Hemorrhagic presentation of a meningioma is a rare but potentially devastating event. Early recognition of the potential underlying meningioma as a cause of bleeding followed by rapid appropriate additional imaging is crucial to direct treatment plans to achieve the best outcome.
